# Supplementary material for: Hand grab or noose pole? Evaluating the least stressful practice for capture of endangered Turks and Caicos Rock Iguanas Cyclura carinata
Source: PeerJ. 2024 Apr 18;12:e17171. doi: 10.7717/peerj.17171 (PMC11032654; doi:10.7717/peerj.17171)
Supplement: Supplemental Information 1 — Distribution of Scaled Mass Index in male and female individuals of Cyclura carinata iguanas. A boxplot is superimposed to the violin plots. The violin plots show the probability density of the data at different values. Each boxplot indicates the median value (horizontal black line), the first and third quartile (lower and upper edges of the rectangle) the minimum and maximum of the distribution (lower and upper whiskers) and the outliers (black dots beyond the minimum or maximum). The red dot within the rectangles corresponds to the mean SMI value in males and females respectively. [file peerj-12-17171-s001.docx]

**Supplementary materials**

**Hand grab or noose pole? Evaluating the least stressful practice for capture of endangered Turks and Caicos Rock Iguanas *Cyclura carinata***

Giuliano Colosimo^1*^, Gwyneth Montemuro^2^, Gregory A. Lewbart^3^, Gabriele Gentile^1^, Glenn P. Gerber^4^

^1^Department of Biological Sciences, University of Rome Tor Vergata

^2^St. Matthews University on Grand Cayman

^3^North Carolina State University College of Veterinary Medicine, 1060 William Moore Drive, Raleigh, NC 27607, USA

^4^San Diego Zoo Wildlife Alliance, 15600 San Pasqual Valley Road, Escondido, CA 92027, USA

Corresponding author:

Email: [giuliano.colosimo@uniroma2.it](mailto:giuliano.colosimo@uniroma2.it)

Orcid ID:

- Giuliano Colosimo: 0000-0002-0485-9758
- Gregory A. Lewbart: 0000-0003-0716-1387
- Gabriele Gentile: 0000-0002-1045-6816
- Glenn P. Gerber: 0009-0006-7353-2958


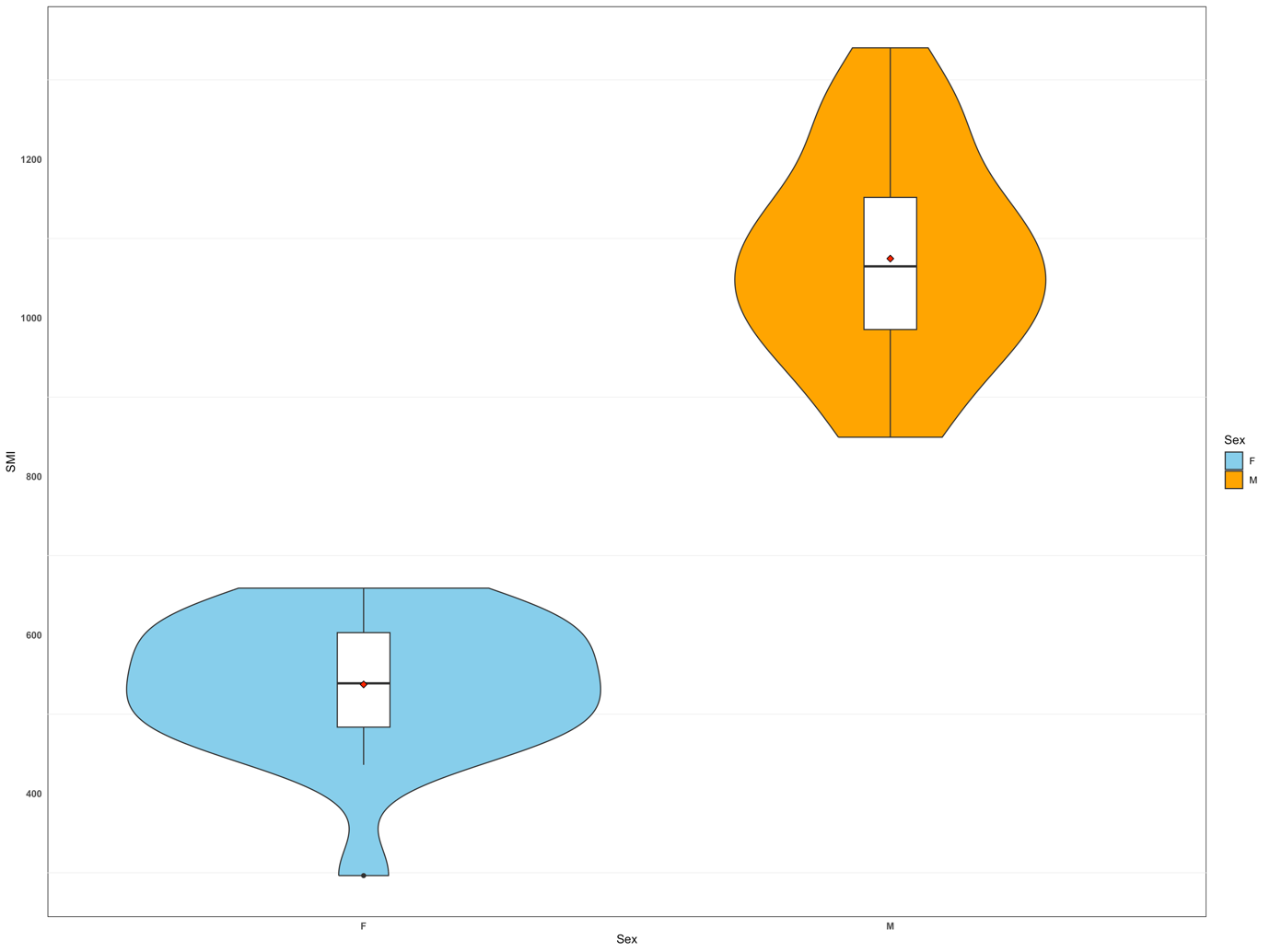


**SM Figure 1** Distribution of Scaled Mass Index in male and female individuals of Cyclura carinata iguanas. A boxplot is superimposed to the violin plots. The violin plots show the probability density of the data at different values. Each boxplot indicates the median value (horizontal black line), the first and third quartile (lower and upper edges of the rectangle) the minimum and maximum of the distribution (lower and upper whiskers) and the outliers (black dots beyond the minimum or maximum). The red dot within the rectangles corresponds to the mean SMI value in males and females respectively.
